# Muscle strength changes and physical activity during gender‐affirming hormone therapy: A systematic review

**DOI:** 10.1111/andr.70058

**Published:** 2025-05-16

**Authors:** Mathilde Kamp Nørlund, Louise Lehmann Christensen, Marianne Skovsager Andersen, Tine Taulbjerg Kristensen, Jan Frystyk, Jonas Mathiesen, Jakob Lindberg Nielsen, Dorte Glintborg

**Affiliations:** ^1^ Faculty of Health Sciences Clinical Institute University of Southern Denmark Odense Denmark; ^2^ Department of Endocrinology Odense University Hospital Odense Denmark; ^3^ Department of Sports Science and Clinical Biomechanics, Muscle Physiology and Biomechanics Research Unit University of Southern Denmark Odense Denmark; ^4^ Department of Clinical Research, Geriatric Research Unit University of Southern Denmark Odense Denmark

**Keywords:** gender‐affirming hormone therapy, muscle strength, physical activity, transgender

## Abstract

**Background:**

Higher muscle strength is associated with improved overall health and lower mortality. Muscle strength changes during gender‐affirming hormone therapy is possibly linked to gender‐affirming hormone therapy modality, age at initiation, gender‐affirming hormone therapy duration, and physical activity.

**Aim:**

To review published literature on muscle strength changes during gender‐affirming hormone therapy.

**Methods:**

Studies were included if they met the PICOS criteria; P: transgender individuals ≥18 years, I: gender‐affirming hormone therapy, C: gender‐affirming hormone therapy‐naïve transgender persons or cisgender controls, O: muscle strength and physical activity in relation to muscle strength, S: prospective cohorts or cross‐sectional.

**Results:**

Fifteen studies with data on 1206 transgender persons (722 transmasculine persons, median age 23–37 years and 484 transfeminine persons, median age 27–41 years) were included. Prospective design was used in eight out of 15 studies (two out of eight on transmasculine, two out of eight on transfeminine, and four out of eight on both) and seven out of 15 were cross‐sectional (two out of seven on transmasculine, four out of seven on transfeminine, and one out of seven on both). Isometric elbow flexion/extension, lower body strength, and handgrip strength were assessed in one out of 15 studies, four out of 15, and 12 out of 15 studies, respectively. Bias rating was moderate to high.

**Prospective studies:**

Masculinizing gender‐affirming hormone therapy resulted in increased (four out of six studies) or unchanged (two out of six studies) muscle strength, while feminizing gender‐affirming hormone therapy resulted in decreased (three out of six studies) or unchanged (three out of six studies) muscle strength. Muscle strength changes mainly occurred during the first year after initiating gender‐affirming hormone therapy and age at initiation had no impact.

**Cross‐sectional studies:**

Transmasculine and transfeminine persons had higher strength compared with cisgender women, but lower strength than cisgender men. Physical activity was unchanged during gender‐affirming hormone therapy in five out of prospective studies, while transfeminine persons were less physically active than cisgender men in five out of five prospective studies.

**Conclusion:**

Muscle strength appeared to increase during masculinizing gender‐affirming hormone therapy and decrease during feminizing gender‐affirming hormone therapy, whereas physical activity was unchanged. Given high risk of bias, more research is necessary. Improving transgender care requires engagement of transgender persons in physical activity.

## 1 INTRODUCTION

Transgender persons experience incongruence between their gender identity and assigned sex at birth. Transmasculine (TM) persons are assigned female sex at birth but identify as men, whereas transfeminine (TF) persons are assigned male sex at birth but identify as women.^1^ Transgender persons may wish to initiate gender‐affirming hormone therapy (GAHT) to align their appearance with their gender identity.^2^ GAHT is divided into masculinizing and feminizing GAHT, where masculinizing GAHT consists of testosterone therapy and feminizing GAHT consists of estradiol therapy often combined with antiandrogens.^2^ Antiandrogens include cyproterone acetate, spironolactone, or gonadotropin‐releasing hormone agonists (GnRH‐a).^3^ GAHT doses are titrated until sex‐hormone levels correspond to the reference range of the identified gender.^2^ The average age at GAHT initiation is around 23 and 26 years for TM and TF persons, respectively, in European study cohorts.^4^, ^5^ GAHT is expected to be lifelong to maintain mediated physiological changes, which underlines the importance of understanding both positive and negative health outcomes of GAHT.^1^

Muscle strength is an important predictor of physical health and higher muscle strength is associated with a lower risk of all‐cause mortality.^6^, ^7^ The anabolic sex‐hormone testosterone increases muscle strength and muscle mass and decreases fat mass.^8^ In cisgender men, it is thoroughly demonstrated that lack of testosterone is associated with lower muscle strength, lower muscle mass, and higher fat mass.^9^ Lean body mass (LBM) can be applied as an estimate of muscle mass and LBM is associated with muscle strength, which indicates that muscle strength will change in the same direction as LBM.^10^, ^11^ However, hypogonadal cisgender men receiving isolated testosterone therapy had unchanged muscle strength despite significantly increased LBM,^12^, ^13^, ^14^ while testosterone therapy combined with strength training resulted in increased muscle strength in hypogonadal cisgender men.^13^ These findings highlight the importance of physical activity in translating more muscle mass into functionally relevant muscle strength gains.

The completion of male physiological puberty, before initiating feminizing GAHT, contributes to higher muscle strength and muscle mass in TF persons.^15^ The endogenous secretion of testosterone is most often suppressed as part of feminizing GAHT to ensure the development of secondary female sex characteristics, but testosterone suppression could result in decreased muscle mass in TF persons.^1^ On the other hand, estradiol and progesterone could have beneficial effects on skeletal muscle mass and muscle strength change,^16^ which could cause less decrease in muscle mass and muscle strength during feminizing GAHT. In support of this view, post‐menopausal women on estrogen replacement therapy exhibit greater muscle strength compared with women without estrogen replacement therapy^17^ and post‐pubertal girls have higher muscle strength than prepubertal girls.^18^

Many factors influence the effect of GAHT on muscle strength changes, for example, physical activity. More physical activity has been positively associated with increased skeletal muscle strength and muscle power,^19^ while a reduction in physical activity leads to decreased muscle strength.^20^, ^21^, ^22^ Transgender persons participate less in sports and physical activity than cisgender men/women,^23^, ^24^ which may be due to the absence of an inclusive environment, limited social support, and other barriers relating to gender identity (e.g., changing facilities).^25^ Thus, it could be hypothesized that physical activity of transgender persons may influence their muscle strength adaptions to GAHT. However, to the best of our knowledge, there is no systematic review of the possible modifying effect of physical activity on muscle strength changes in transgender populations.

### 1.1 Objective

This systematic review aimed to evaluate changes in muscle strength during GAHT compared with GAHT‐naïve transgender persons or cisgender persons. We further explored the impact of GAHT modality (masculinizing or feminizing), age at GAHT initiation, duration of GAHT, and if changes in physical activity predicted changes in muscle strength.

## 2 METHODS

### 2.1 Eligibility criteria

We made a predefined PICOS (Population, Intervention, Comparison, Outcome, and Study design) prior to the literature search according to the Preferred Reporting Items for Systematic Reviews and Meta‐Analyses (PRISMA) guidelines.^26^ The population was TM and TF persons ≥18 years old who were GAHT‐naïve and approved to initiate GAHT or were already undergoing GAHT at a specialized gender identity clinic. The intervention was GAHT. There was no restriction on GAHT duration, administration form, or intervals between GAHT administration. The comparison had to be GAHT‐naïve transgender persons or cisgender persons, if control groups were included. The outcome was muscle strength, reported as a primary or secondary outcome, but we also aimed to explore physical activity in relation to muscle strength changes. All assessment methods of the outcomes were accepted. The study design had to be prospective or cross‐sectional. The written language had to be English, and the publication date had no restrictions. This review was registered in PROSPERO (ID: CRD42024536256).

### 2.2 Information sources and search

We searched the databases PubMed, EMBASE (Ovid), MEDLINE (Ovid), and Web of Science on December 10, 2024. The predefined PICO defined the search clusters: population (transgender persons), intervention (GAHT), and outcome (muscle strength or physical activity). The search terms were divided by “OR” and the clusters were combined with “AND.” When possible, the search terms were included as MESH‐terms in PubMed and as auto‐exploded terms (exp/) in MEDLINE and EMBASE to conduct a database‐specific search. The abbreviation “TS” for topic search was used in Web of Science to ensure the search terms were present in the title, abstract or keywords. The full search string is presented in Appendix A.

### 2.3 Study selection

The database search results were exported and uploaded to EndNote and Covidence (2024), where duplicates were automatically removed. Two authors (M. K. N. and J. L. N.) conducted the screening process. First, the title and abstract were screened for eligibility based on the inclusion criteria, and second, the remaining articles underwent a full‐text screening. Reference lists of the included studies were manually searched aiming at identifying further articles for screening.

### 2.4 Data extraction

Data extraction included a general characteristic of the study (authors, year of publication, country, study design, and follow‐up period), the study population (sample size, gender identity, control group, age at GAHT initiation, GAHT administration and dose, sex‐hormone levels), the outcome assessment method (muscle strength tests and physical activity assessments), and outcome results. If studies involved more than one strength measurement, data were extracted using the following order: isometric strength and dynamic strength. PlotDigitizer (Porbital 2025) was used to extract numerical data (mean ± standard deviation) from studies, where muscle strength measurements were only available in graphs.^4^, ^27^

### 2.5 Risk of bias assessment

The Newcastle‐Ottawa (NOS) quality assessment tool^28^ was used for risk of bias assessment, as NOS is designed to rate non‐randomized study designs. The original NOS was used for prospective studies, while an adapted version was used for cross‐sectional studies (Appendix B). Each study was assessed across three categories: selection of the study group, comparability, and outcome, with each category compromising subcategories, each eligible to receive stars upon meeting subcategory‐specific low‐bias criteria. The overall risk of bias in the studies was rated using the threshold of low risk of bias (7–9 stars), moderate risk of bias (4–6 stars), and high risk of bias (0–3 stars). Studies were assigned a star in “representativeness of the exposed cohort” if the study population were transgender persons approved for GAHT at a gender identity clinic. Prospective and cross‐sectional studies obtained a star in “selection of the non‐exposed cohort” if the control group was GAHT‐naïve transgender persons, for example, waiting list participants, or cisgender persons. NOS states that only secure records or structured interviews would merit a star in “ascertainment of exposure,”^28^ but in the present study a star was given if GAHT was confirmed by sex‐hormone level assessment. Prospective studies needed a baseline outcome assessment to demonstrate “outcome of interest was not present at start of study.” Studies would receive a star for “comparability” if the control group was matched (e.g., age, BMI, and physical activity). Low risk in “assessment of outcome” required independent blind assessment or record linkage. Prospective studies’ follow‐up duration had to be ≥3 months in “follow‐up long enough for outcomes to occur,” as it has been suggested to be the minimum duration for muscle strength adaptions in both TM and TF persons.^29^ The prospective studies needed a follow‐up rate ≥80% to receive a star in “Adequacy of follow‐up of cohorts.”

Cross‐sectional studies received stars in “sample size” if they included >39 transgender persons in each group, based on a sample size estimation from a protocol article on TM persons on muscle strength.^30^ A star was assigned in “non‐respondent” if comparability between respondent and non‐respondents characteristics was established, and the response rate was satisfactory or if there was statistically adjusted for differences between the groups. Cross‐sectional studies would receive a star in “statistical test” if the statistical test were clearly described and appropriate, including confidence intervals and *p* values. Corresponding authors were contacted in case of missing information.

## 3 RESULTS

### 3.1 Study selection

**FIGURE 1:**
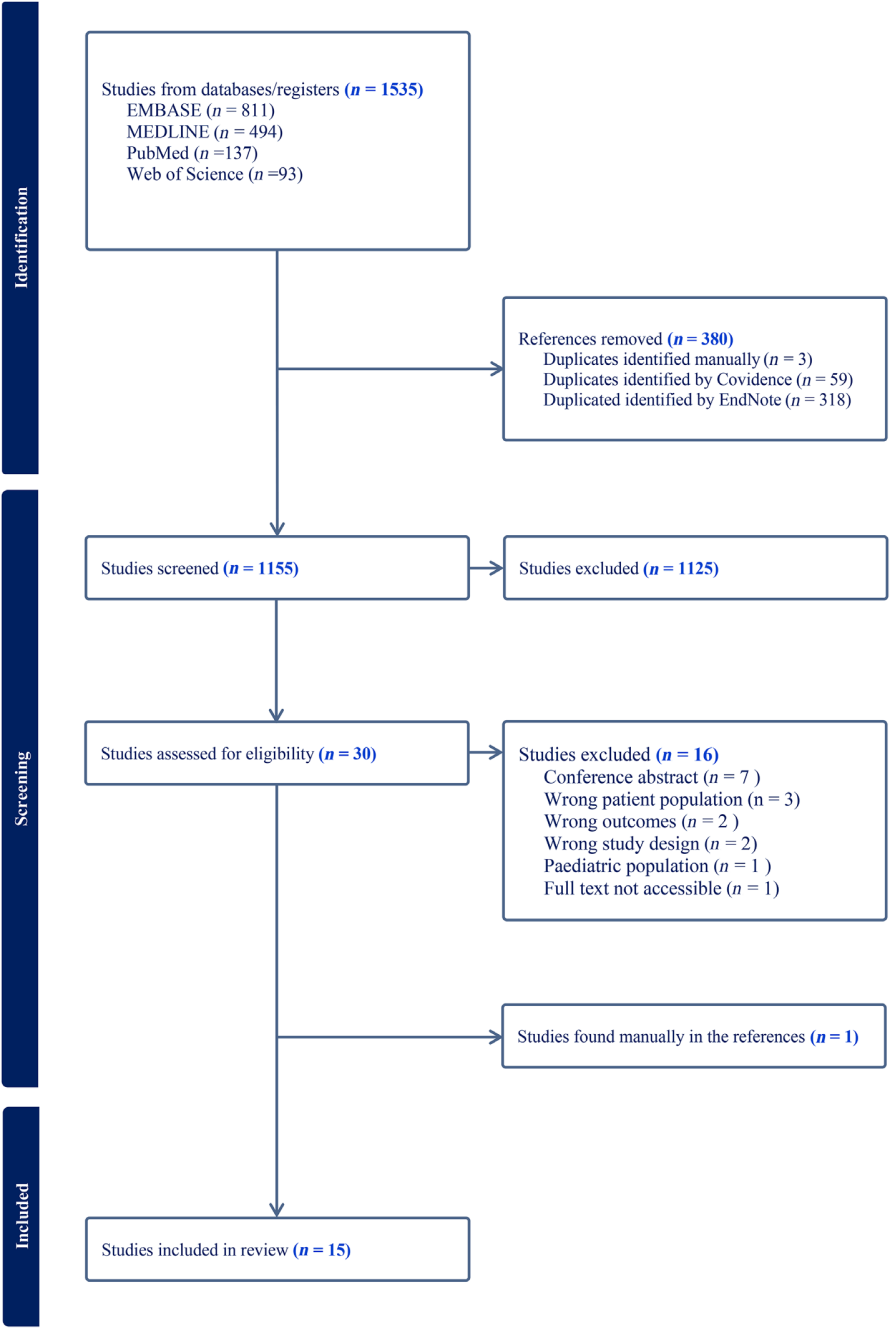
PRISMA flowchart.

The study selection process according to PRISMA‐guidelines are presented in Figure 1. The initial search retrieved 1535 results, of which 380 were duplicates. EndNote identified 318 duplicates, Covidence identified 59 duplicates, and three duplicates were identified manually. Thus, a total of 1155 articles were screened by title and abstract, resulting in 1125 studies being excluded, leaving 30 articles for full‐text screening. An additional 16 studies were deemed ineligible due to incorrect outcome, wrong patient population, inclusion of patients <18 years old, utilizing an unsuitable study design, being conference abstract or having an inaccessible full text. One prospective study was found when manually searching references of relevant studies.^31^ A total of 15 articles were eligible and included in the review. One of the included studies recruited participants through social media, requiring transgender persons to have undergone GAHT for at least 1 year. The participants received GAHT at both specialized and private gender identity clinics. However, upon contacting the authors, they could not provide information regarding the distribution of participants treated at specialized versus private clinics.^32^ Nevertheless, the study was deemed eligible for inclusion.

### 3.2 Characteristics of included studies

Characteristics of the 15 included studies are presented in Table 1, including data from 1206 transgender persons (722 TM persons and 484 TF persons). Sample sizes ranged from 11 to 527 participants. Age in TM and TF persons ranged from 23–37 and 27–41 years, respectively. Studies were published between year 2008 and 2024 and originated from Europe,^4^, ^27^, ^31^, ^32^, ^33^, ^34^, ^35^, ^36^, ^37^, ^38^ South America,^39^, ^40^ and Asia.^41^, ^42^, ^43^ Five studies^4^, ^31^, ^33^, ^35^, ^37^ were part of the “European Network for Investigation of Gender Incongruence” (ENIGI), a multicenter prospective cohort study from Belgium, Norway, the Netherlands, and Germany. The first publication regarding muscle strength was from Van Caenegem (2012),^37^ where TM persons from the Ghent cohort were compared with cisgender women. Afterward, Van Caenegem (2015)^33^ and Van Caenegem (2015)^35^ published data regarding TM and TF persons from the Ghent cohort. One year later, Auer (2016)^31^ also published data from the Ghent cohort. However, they only included a subgroup of TM and TF persons focusing on metabolomics rather than muscle strength changes. Finally, Scharff (2019)^4^ was a multicenter prospective cohort study including both TM and TF persons from the Amsterdam, Ghent, and Oslo cohort. Furthermore, Lapauw (2008)^36^ also included participants from the Ghent cohort, but data were published before the establishment of ENIGI in 2010. Therefore, results from participants in Van Caenegem (2012),^37^ Van Caenegem (2015),^33^ Van Caenegem (2015),^35^ and Auer (2016)^31^ were expected to be included in Scharff (2019).^4^ Two other studies^27^, ^38^ originated from the same center where Lundberg (2024)^38^ was a continuation of the study by Wiik (2020).^27^

**TABLE 1:** Characteristics of the 15 included studies.

Prospective cohort studies, *n* = 8									
Author (year)	Country	Design and follow‐up	Participants number		Participants age (years)	GAHT	Assessments		Results
			TM	TF		Route and dose	Muscle strength	Physical activity	
Lundberg et al. (2024)	Sweden	Pre‐GAHT, 1 and 5–6 year	T0, *n* = 17 1y, *n* = 16 6y, *n* = 12	T0, *n* = 16 1y, n = 14 5y, *n* = 7	TM: 25 ± 5 TF: 28 ± 5	TM: 1 g T‐undecanoate i.m. TF: 1–2 mg EV gel, 100–200 μ q estradiol patch, 4–8 mg oral EV, or 80 mg EV i.m. Both GnRH‐a before GAHT	Isometric knee extensor peak torque (Nm)	IPAQ‐SF modified to Swedish context	TM: T0: 174.0 ± 43.0 1y: 192.0 ± 39.0 +10.3%^*^ 6y: 206.0 ± 35.0 +16.7%^*^ TF: T0: 238.0 ± 71.0 1y: 247.0 ± 81.0 +3.8% (NS) 5y: 220.0 ± 68.0 −7.3% (NS) Physical activity unchanged
Tominaga et al. (2024)^a^	Japan	Pre‐GAHT and at follow‐up visits every 3 months during the first year, then annually up to the 10th year post‐GAHT	T0, *n* = 291 1y, *n* = 244 5y, *n* = 94 10y, *n* = 12	–	TM: 25 ± 6	125–250 mg T esters i.m.	Handgrip strength, dominant hand (kg)	NA	Low dose/high dose: T0: 28.9 ± 4.7/28.0 ± 4.6 1y: 30.8 ± 4.9/31.5 ± 5.3 (NS) 5y: 31.2 ± 4.7/21.7 ± 4.8 (NS) 10y: 30.2 ± 3.9/NA (NS).
Yun et al. (2021)	Korea	Pre‐GAHT and 6 months	–	11	TF: 29 ± 8	2–4 mg oral EV (*n* = 10) or 5–10 mg EV i.m. (*n* = 1) 100 mg SPN (*n* = 5) or 25–50 mg CPA (*n* = 6)	Handgrip strength (kg), left and right hand	NA	Left hand: T0: 31.5 ± 5 6 months: 29.9 ± 7.0 −6.2% (NS) Right hand: T0: 31.8 ± 6.0 6 months: 30.1 ± 6.0 –7.7%^*^
Wiik et al. (2020)^b^	Sweden	Pre‐GAHT, 1, 4, and 12 months	12	11	TM: 25 ± 5 TF: 27 ± 4	TM: 1 g T‐undecanoate i.m. TF: 1–2 mg EV gel, 100–200 μ q estradiol patch, 4–8 mg oral EV, or 80 mg EV i.m. Both GnRHA before GAHT	Isometric and isokinetic knee extensor and flexor peak torque (Nm)	Questionnaires (not specified)	TM, extensor/flexor^†^: T0: 172.0 ± 39.4/76.6 ± 16.1 1 month: 170.0 ± 35.2/79.0 ± 17.7 4 months: 186.5 ± 31.1/86.3 ± 13.7 1y: 192.7 ± 37.3 + 12.0%^*^/94.3 ± 17.7 +23.2%^*^ TF, extensor/flexor^†^: T0: 240.4 ± 47.7/100.0 ± 17.0 1 month: 238.3 ± 39.4/103.8 ± 17.7 4 months: 242.5 ± 43.5/100.0 ± 19.4 1y: 242.5 ± 43.5 + 0.9%/101.6 ± 17.0 +1.6% (NS) Physical activity unchanged
Scharff et al. (2019)^c^	The Nether‐lands, Norway, and Belgium (ENIGI)	Pre‐GAHT, 3, 6, 9, and 12 months	278	249	TM: 23 (20–30) TF: 28 (23–40)	TM: 50 mg T gel (*n* = 52), 250 mg T‐esters i.m. (*n* = 81) or 1 g T‐undecanoate i.m. (*n* = 130) TF: 2–4 mg oral EV and 50 mg CPA (*n* = 134), 100 μ q estradiol patch and 50 mg CPA (*n* = 86)	Handgrip strength (kg)	Only reported for subgroups	TM: T0: 33.1 ± 6.5 3 months: 36.0^†^ 6 months: 38.3^†^ 9 months: 38.4^†^ 1y: 39.2 ± 6.8 +18.4%^*^ TF: T0: 41.8 ± 8.9 3 months: 40.4^†^ 6 months: 40.5^†^ 9 months: 40.6^†^ 1y: 40.0 ± 8.9 –4.3%^*^
Auer et al. (2016)	The Nether‐lands, Norway, Belgium, and Germany (ENIGI)	Pre‐GAHT and 12 months	20	20	NA.	TM: 1 g T‐undecanoate, 5 mg lynestrenol (*n* = 6) TF: 4 mg oral EV and 50 mg CPA (*n* = 13), 100 μ q estradiol patch and 50 mg CPA (*n* = 7)	Handgrip strength (kg)	Baecke's questionnaire	TM: T0: 30.2 ± 7.3 1y: 32.4 ± 6.1 (NS) TF: T0: 41.7 ± 8.9 1y: 41.9 ± 6.1 (NS) Physical activity unchanged
Van Caenegem et al. (2015)	Belgium (ENIGI)	Pre‐GAHT and 12 months	T0, *n* = 23 1y, *n* = 23	–	TM: 27 ± 9 CW: 27 ± 9	1 g T‐undecanoate i.m. (*n* = 22) or T‐esters i.m. (*n* = 1)	Handgrip strength (N/kg), dominant hand	Baecke's questionnaire	TM: T0: 30.0 ± 6.0 1y: 35.0 ± 5.0 +18.2%^*^ CW: T0: 32.0 ± 4.0 1y: 31.0 ± 4.0 –1.2% (NS) Physical activity unchanged
Van Caenegem et al. (2015)	Belgium (ENIGI)	Pre‐GAHT, 12, and 24 months	–	T0, *n* = 49 1y, *n* = 44 2y, *n* = 29	TF: 33 ± 12 CW: 33 ± 12	4 mg oral EV and 50 mg CPA (*n* = 34) or 100 μq estradiol patch and 50 mg CPA (*n* = 15)	Handgrip strength (N/kg), dominant hand	Baecke's questionnaire	TF: T0: 42.0 ± 9.0 1y: 39.0 ± 9.0 −7.1%^*^ 2y: 38.0 ± 10.0 –10.3%^*^ CM: T0: 49.0 ± 6.0 Physical activity unchanged in TF. TF was less physically active than CM.

Case–control studies, *n* = 7											
Author (year)	Country	Design GAHT duration	Participants number				Participants age (years)	GAHT	Assessments		Results
			TM	TF	CM	CW		Route and dose	Muscle strength	Physical activity	
Saitong et al. (2024)	Thailand	TF with orchiectomy GAHT duration 8.7 ± 3.1 years TF without orchiectomy GAHT duration 10.1 ± 4.0 years	–	30 (15 had undergone oriectomy)	15	15	TF with oriectomy: 30 ± 4 TF without oriectomy: 27 ± 4	4 mg oral EV or 2–6 mg EV gels with either oriectomy (*n* = 15) or 50 mg CPA (*n* = 15)	Isokinetic knee and elbow extension and flexion peak torque (Nm)	GPAQ	TF with oriectomy: Elbow extension/flexion: 27.2 ± 5.4^*^ vs. CM /21.5 ± 5.7^*^ vs. CM Knee extension/flexion: 112.4 ± 32.3^*^ vs. CM/48.9 ± 11.6^*^ vs. CM. TF without oriectomy: Elbow extension/flexion: 29.1 ± 5.2^*^ vs. CM/21.3 ± 5.0^*^ vs. CM Knee extension/flexion: 123.5 ± 23.9^*^ vs. CM/51.6 ± 6.0^*^ vs. CM Physical activity similar between groups
Amador et al. (2024)	Spain	Post‐GAHT (mean GAHT duration 37 months (median 29 months)	–	37	34	34	TF: 29 (20–38) CM: 29 (21–36) CW: 29 (20–42)	Oral EV and CPA	Handgrip strength (kg), left and right hand	NA	TF, right hand: 26.3 ± 6.2 TF, left hand: 24.8 ± 6.0 CW, sarcopenia cutoff: < 16.0 kg. CM, sarcopenia cutoff: < 27.0 kg.
Hamilton et al. (2024)	United Kingdom	Post‐GAHT TF GAHT duration 6 ± 4 years TM GAHT duration 4 ± 5 years	12 (athletes, min. physical training 3 times pr. week)	23 (athletes, min. physical training 3 times pr. week	19	21	TM: 34 ± 7 TF: 34 ± 10 CM: 37 ± 9 CW: 30 ± 9	NA	Handgrip strength (kg), right hand	4 session per week—training dairies	TM: 38.8 ± 7.5^*^ vs. TF and CM. TF: 40.7 ± 6.8 CM: 45.7 ± 6.9 CW: 34.3 ± 3.8^*^ vs. TF and CM. Physical activity similar between groups
Alvares et al. (2022)	Brazil	Post‐GAHT GAHT duration 14.4 ± 3.5 years	–	15	14	13	TF: 34 ± 5 CM: 37 ± 4 CW: 36 ± 4	Oral EV (*n* = 5), estradiol gel (*n* = 3) or conjugated estrogen (*n* = 7) and CPA (*n* = 11)	Handgrip strength (kg), right hand	IPAQ‐SF	TF: 35.3 ± 5.4^*^ vs. CW. CM: 48.8 ± 6.7 CW: 29.7 kg ± 3.6^*^ vs. CM. Physical activity similar between groups
Andrade et al. (2021)	Brazil	Post‐GAHT Mean GAHT duration 24 months (range: 10–28 months)	19	–	19	–	TM: 24 ± 3 CM: 24 ± 3	200 mg T i.m. or 50 mg subcutaneously	Handgrip strength (kg), dominant hand	IPAQ‐SF	TM: 28.8 ± 7.5 CM: 40.3 ± 8.0^*^ vs. TM Physical activity similar between groups
Van Caenegem et al. (2012)^d^	Belgium (ENIGI)	Post‐GAHT Mean GAHT duration 9.9 years (range: 3.2–27.5 years)	TM receiving GAHT: 50 TM not receiving GAHT: 16	–	–	CW1: 50 CW2: 16	TM with GAHT: 37 ± 8 CW1: 38 ± 8 TM without GAHT: 28 ± 7 CW2: 26 ± 7	T‐esters i.m. (*n* = 35), 1 g T‐undecanoate i.m. (*n* = 7), or 50 mg T transdermal (*n* = 8)	Handgrip strength (kg), dominant hand	Baecke's questionnaire	TM with GAHT: 37.0 ± 6.0 TM without GAHT: 33.0 ± 7.0 CW1: 29.0 ± 5.0^*^ vs. TM with GAHT. CW2: 32.0 ± 5.0 Physical activity similar between groups
Lapauw et al. (2008)	Belgium	Post‐GAHT Mean GAHT duration 8 years (range: 4–20 years)	–	23	46	–	TF: 41 ± 7 CM: 40 ± 7	20–50 μq ethinyl estradiol (*n* = 8), 2 mg EV (*n* = 10), 1.25 mg conjugated estrogens (*n* = 2) or transdermal estradiol gel (*n* = 3)	Handgrip strength (kg), dominant hand Isokinetic peak torque of upper arm and leg (Nm)	Baecke's questionnaire	TF: Grip strength: 41.0 ± 8.0^*^ vs. CM. Peak torque, UE: 38.0 ± 13.0^*^ vs. CM Peak torque, LE: 150.0 ± 49.0^*^ vs. CM CM: Grip strength: 53.0 ± 8.0 Peak torque, UE: 57.0 ± 14.0 Peak torque, LE: 200.0 ± 44.0 TF had lower sports‐related physical activity than CM

Studies are listed with cohort studies first, followed by cross‐sectional studies, in descending order of publication date. Data are presented as mean ± standard deviation or as median (interquartile range 25–75%), as reported in the original articles.

Abbreviations: CM, cisgender men; CPA, cyproterone acetate; CS, cross‐sectional study design; CW, cisgender women; EV, estradiol valerate; GAHT, gender affirming hormone therapy; GnRHA, gonadotropin releasing hormone antagonist; GPAQ, global physical activity questionnaire; i.m., intramuscular; IPAQ‐SF, international physical activity questionnaire (short form); kg, kilograms; LE, lower extremity; NA, not available; Nm, Newton·meter; N/kg, Newton per kilogram; NS, non‐significant changes; Pros., prospective; SPN, spironolactone; T, testosterone; T0, baseline visit; TF, transfeminine persons; TM, transmasculine persons; UE, upper extremity; y, year follow‐up.

^a^ Selected temporal data presented.

^b^ We only focused on isometric strength in this systematic review.

^c^ Authors were contacted to obtain missing data on the number of participants at various follow‐up time points, but we received no answer.

^d^ Cisgender women 1 (CW1): control group to transmasculine with GAHT, cisgender women 2 (CW2): control group to transmasculine without GAHT.

* Data reported as significant (*p* < 0.05) in the original articles.

^†^ PlotDigitizer were used to convert graphs to estimated numerical mean values.

A prospective study design was used in eight out of 15 studies that investigated the effect of GAHT on muscle strength changes in GAHT‐naïve persons after GAHT initiation with different follow‐up durations (6 months to 10 years). TM persons were assessed in two out of eight prospective studies,^33^, ^42^ TF persons were assessed in two out of eight prospective studies,^35^, ^41^ and both TM and TF persons were assessed in four out of eight prospective studies (TM and TF results were reported separately).^4^, ^27^, ^31^, ^38^ Transgender persons were compared with cisgender men/women in three out of eight prospective studies,^27^, ^33^, ^35^ while no comparisons with control groups were made in the remaining five out of eight prospective studies.^4^, ^31^, ^38^, ^41^, ^42^

The follow‐up period of the prospective studies was 3, 6, 9, and 12 months,^4^ 6 months,^41^ 1 and 2 years,^35^ 1 year,^27^, ^31^, ^33^ 1, 5, and 6 years,^38^ and every 3 months during the first year of GAHT, then annually up to 10‐years post‐GAHT.^42^

A cross‐sectional study design was used in seven out of 15 studies,^32^, ^34^, ^36^, ^37^, ^39^, ^40^, ^43^ where transgender persons on ongoing GAHT were compared with cisgender men/women of the same or opposite birth‐assigned sex. TM persons were investigated in three out of seven cross‐sectional studies, with comparison with cisgender women in one study^37^ and comparison with cisgender men in two studies.^32^, ^40^ TF persons were investigated in five out of seven cross‐sectional studies, with comparisons with both cisgender men and women in three studies,^34^, ^39^, ^43^ to cisgender men in one study,^36^ and to cisgender women in one study.^32^ None of the studies compared the TM or TF persons with GAHT‐naïve transgender persons.

In cross‐sectional studies, the duration of masculinizing GAHT was 2,^40^ 4,^32^ and 9.9 years^37^ and the duration of feminizing GAHT was 3.1,^34^ 6,^32^ 8,^36^ 8.7 years in TF persons with orchiectomy and 10.1 years in TF persons without orchiectomy,^43^ and 14.4 years.^39^

The method for assessing muscle strength was elbow flexion and extension peak torque in one out of 15 studies,^43^ lower body isometric and/or isokinetic knee extensor peak torque muscle strength in four out of 15 studies,^27^, ^36^, ^38^, ^43^ and handgrip strength in 12 out of 15 studies.^4^, ^31^, ^32^, ^33^, ^34^, ^35^, ^36^, ^37^, ^39^, ^40^, ^41^, ^42^ However, isometric strength has been shown to have the highest methodological reliability^38^, ^44^ and to optimize inter‐study homogeneity, isokinetic strength was not included in this systematic review. Elbow flexion and extension peak torque were registered as the average of the two highest values.^43^ Lower body peak torque was registered as the highest value in one study,^36^ but the method for reporting muscle strength was not given in the remaining two studies assessing lower body peak torque.^27^, ^38^ Handgrip strength was registered as the highest value of three attempts in six out of 12 studies^4^, ^33^, ^35^, ^36^, ^37^, ^40^ and as the mean value of three attempts in five out of 12 studies.^32^, ^34^, ^39^, ^41^, ^42^ The last study measuring handgrip strength did not report how the outcome was registered.^31^

Physical activity was assessed in 11 out of 15 studies. The method for physical activity assessment was questionnaires in 10 out of 11 studies, while the last study required participants to participate in competitive sports or undergo physical training at least three times a week, and self‐report their training intensity for their last four training sessions before study inclusion.^32^ Of the 10 studies using questionnaires, five used Baecke's questionnaire^31^, ^33^, ^35^, ^36^, ^37^ assessing physical activity on yearly and weekly basis differentiated into work, sports, and leisure activities,^45^ while four,^27^, ^38^, ^39^, ^40^ and one study^43^ used the international physical activity questionnaire (IPAQ) and the global physical activity questionnaire (GPAQ), respectively, which documents physical activity over the past 7 days.^46^, ^47^

### 3.3 Risk of bias assessment and homogeneity

Risk of bias assessment for the prospective studies are shown in Table 2 and for cross‐sectional studies are shown in Table 3. No study was rated “low risk of bias,” 11 out of 15 studies were rated “moderate risk of bias,” and four out of 15 studies were rated “high risk of bias.”

**TABLE 2:** Risk of bias—Newcastle‐Ottawa scale for cohort studies.

Study	Selection (S)				Comparability (C)	Outcome (O)			Total (T)			
	Representativeness of exposed cohort True = Recruitment from gender clinic	Selection of non‐exposed cohort	Ascertainment of exposure to GAHT	Outcome assessment prior to treatment	Comparability	Assessment of outcome	Follow‐up duration	Adequacy of follow up	S	C	O	T
Lundberg et al. (2024)	True^a^	None	Sex‐hormone levels^a^	Assessed 4 weeks after GnRH treatment^a^	Cisgender men and women	No description	Yes: 5–6 years^a^	Follow up rate <80% and no description of those lost	3	0	1	4
Tominaga et al. (2024)	True^a^	None	Sex‐hormone levels^a^	Yes^a^	None	No description	Yes: 10 years^a^	No description	3	0	1	4
Yun et al. (2021)	Somewhat representative: TF from gender clinics but excluded if psychological vulnerability^a^	None	None	Yes^a^	None	No description	Yes: 6 months^a^	No description	2	0	1	3
Wiik et al. (2020)	True^a^	None	Sex‐hormone levels^a^	Assessed 4 weeks after GnRH treatment^a^	Cisgender men and women	No description	Yes: 12 months^a^	Complete follow up—all subjects accounted for^a^	3	0	2	5
Scharff et al. (2019)	True^a^	None	Sex‐hormone levels^a^	Yes^a^	None	No description	Yes: 3, 6, 9, and 12 months^a^	Subjects lost to follow up unlikely to introduce bias^a^	3	0	2	5
Auer et al. (2016)	True^a^	None	Sex‐hormone levels^a^	Yes^a^	None	No description	Yes: 12 months^a^	No description	3	0	1	4
Van Caenegem et al. (2015)	True^a^	Cisgender women	Sex‐hormone levels^a^	Yes^a^	Cisgender women matched for age	No description	Yes: 12 months^a^	Complete follow up—all subjects accounted for^a^	3	0	2	5
Van Caenegem et al. (2015)	True^a^	Cisgender men	Sex‐hormone levels^a^	Yes^a^	Cisgender men matched for age	No description	Yes: 12 and 24 months^a^	Complete follow up—all subjects accounted for^a^	3	0	2	5

The risk of bias was rated as follows: 0–3 stars: high risk of bias, 4–6 stars: moderate risk of bias, 7–9 stars: low risk of bias.

Abbreviations: TF, transfeminine persons; TM, transmasculine persons; GAHT, gender affirming hormone therapy.

^a^ Allocated star following the guidelines of NOS.

**TABLE 3:** Risk of bias—Newcastle‐Ottawa scale for cross‐sectional studies.

Study	Selection (S)				Comparability (C)	Outcome (O)		Total (T)			
	Representativeness of exposed cohort True = recruitment from gender clinic.	Sample size	Non‐respondents	Ascertainment of exposure to GAHT	Confounding factors are controlled	Assessment of outcome	Statistical test	S	C	O	T
Saigon et al. (2024)	True^a^	Not justified	No description	Sex‐hormone levels^a^	Study controls for age and physical activity^b^	No description	Appropriate^a^	2	2	1	5
Amador et al. (2024)	True^a^, ^,c^	Not justified	No description	None	No controlling for confounders	No description	Appropriate^a^	1	0	1	2
Hamilton et al. (2024)	Somewhat representative, from both gender clinic and private clinic^a^, ^,c^	Not justified	No description	Sex‐hormone levels^a^	No controlling for confounders	No description	Appropriate^a^	2	0	1	3
Alvares et al. (2022)	True^a^	Not justified	No description	Sex‐hormone levels^a^	Study controls for age and physical activity^b^	No description	Appropriate^a^	2	2	1	5
Andrade et al. (2021)	Somewhat representative: TM from outpatient clinic^a^	Not justified	No description	Sex‐hormone levels^a^	No controlling for confounders	No description	Appropriate^a^	2	0	1	3
Van Caenegem et al. (2012)	True^a^	Justified^a^	No description	Sex‐hormone levels^a^	Study controls for age^a^	No description	Appropriate^a^	3	1	1	5
Lapauw et al. (2008)	True^a^	Not justified	No description	Sex‐hormone levels^a^	Study controls for age and height^a^	No description	Appropriate^a^	2	1	1	4

The risk of bias was rated as follows: 0–3 stars: high risk of bias, 4–6 stars: moderate risk of bias, 7–9 stars: low risk of bias.

Abbreviations: TF, transfeminine persons; TM, transmasculine persons; GAHT, gender affirming hormone therapy.

^a^ allocated 1 star following the guidelines of NOS.

^b^allocated 2 stars following the guidelines of NOS.

^c^ Author information, additional information not included in the original published paper.

In “representativeness of exposed cohort,” all studies obtained one star, as one inclusion criterion was recruitment at a specialized gender identity clinic. However, three out of 15 studies were evaluated as “somewhat representative,” as they excluded transgender participants with psychological vulnerabilities, which is prevalent in transgender persons,^48^ or included patients from both private clinics and gender identity clinics.^32^ In “selection of the non‐exposed cohort,” none of the studies received a star as the control group was not drawn from the same community as the exposed cohort. Van Caenegem (2012)^37^ included a group of GAHT‐naïve TM persons, but comparisons were made with the control group of cisgender women rather than the included TM persons on GAHT. “Assessment of outcome” was rated “poor” in all studies, as there was no description of who performed the strength tests and whether the investigators were blinded to GAHT modality or previous results in the strength tests. “Ascertainment of exposure” was determined by sex‐hormone levels in 13 out of 15 studies,^4^, ^27^, ^31^, ^32^, ^33^, ^35^, ^36^, ^37^, ^38^, ^39^, ^40^, ^42^, ^43^ while two out of 15 did not report sex‐hormone levels.^34^, ^41^ Authors were contacted regarding this matter, but we received no response.

Muscle strength and physical activity assessment methods varied across the included studies, introducing heterogeneity in the results. In 14 out of 15 studies,^4^, ^27^, ^31^, ^33^, ^34^, ^35^, ^36^, ^37^, ^38^, ^39^, ^40^, ^41^, ^42^, ^43^ TM and TF persons achieved sex‐hormone levels within the reference range of their identified gender.^2^ In Hamilton (2024),^32^ TF persons demonstrated estradiol levels (mean ± SD, 742.4 ± 801.9 pmol/L) exceeding the reference range of cisgender women.

### 3.4 GAHT administration and doses

GAHT administration, doses, and therapy intervals varied across the studies.

TM persons were treated with testosterone undecanoate, 1000 mg intramuscular injection every 12 weeks in six studies.^4^, ^27^, ^31^, ^33^, ^37^, ^38^ Testosterone esters, 125–250 mg intramuscular injection every 2–3 weeks was used in five studies,^4^, ^33^, ^37^, ^40^, ^42^ or 50 mg subcutaneously administration weekly in one study.^40^ Transdermal testosterone gel 50 mg per day was used in two studies.^4^, ^37^

TF persons were treated with estradiol valerate, 2–4 mg tablets per day in nine studies.^4^, ^27^, ^31^, ^34^, ^35^, ^36^, ^38^, ^39^, ^41^ Estradiol valerate intramuscular injection every 2–4 weeks was used in three studies.^27^, ^38^, ^41^ Transdermal 17 β ‐estradiol gel 0.5–1.5 mg per day was used in seven studies.^4^, ^27^, ^31^, ^35^, ^36^, ^38^, ^39^ Estradiol patches 100 μ q/24 h twice a week were used in five studies.^4^, ^27^, ^31^, ^35^, ^38^ Conjugated estrogen 1.25 mg per day or ethinyl estrogen 25–50 μ g per day were used in two studies.^36^, ^39^ Estradiol therapy was combined with the antiandrogen cyproterone acetate 50–100 mg per day in seven studies^4^, ^31^, ^34^, ^35^, ^36^, ^39^, ^41^ and spironolactone 100 mg per day in one study.^41^ GnRH analogues were used in GAHT‐naïve TM and TF persons 3–4 weeks prior to GAHT in two studies.^27^, ^38^ One study did not report the type of GAHT administration.^32^ Upon contacting the authors, they disclosed that the GAHT regimens were highly heterogeneous, involving cyproterone acetate, GnRH‐a, subcutaneous injections or patches. Study protocols for GAHT varied (e.g., different administration intervals, doses, and administration methods) introducing additional heterogeneity that precluded the possibility of conducting a meta‐analysis.

### 3.5 Muscle strength

#### 3.5.1 Masculinizing GAHT

##### Prospective studies

Muscle strength increased significantly in four out of six prospective studies in GAHT‐naïve TM person after masculinizing GAHT,^4^, ^27^, ^33^, ^38^ while muscle strength was unchanged in two out of six studies (Table 1).^31^, ^42^ In Wiik (2020),^27^ lower body isometric torque increased 12% for knee extension and 26% for knee flexion after 12 months. In Lundberg (2024),^38^ knee extension increased 18% from baseline to 6 years post‐GAHT, making it an additional increase of 6% from 1 to 5 years post‐GAHT. Handgrip strength increased 18.4% from baseline to 12‐months follow‐up in Scharff (2019),^4^ with 49% of the increase taking place within the first 3 months. Similarly, handgrip strength increased by 18.2% from baseline to 12 months follow‐up in Van Caenegem (2015).^33^ In Auer (2016)^31^ and Tominaga (2024),^42^ handgrip strength did not increase significantly during 12 months of GAHT. However, Tominaga (2024)^42^ reported a trend for increased handgrip strength during the first 12 months of treatment, which then stabilized over the subsequent 9 years, extending to 10‐years post‐GAHT initiation.

Differences in muscle strength changes were investigated between various age groups (<25, 25–40, and >40 years old) in Scharff (2019).^4^ Changes in handgrip strength after 12 months of GAHT were non‐significant between different age groups.

##### Cross‐sectional studies

In two cross‐sectional studies comparing TM persons using masculinizing GAHT with cisgender men, handgrip strength was lower in TM persons after 2 and 6 years of masculinizing GAHT compared with cisgender men (Table 1).^32^, ^40^ In Hamilton (2024),^32^ TM persons’ handgrip strength was 38.8 ± 7.5 kg compared with 45.7 ± 6.9 kg in cisgender men, while in Andrade (2022),^40^ TM persons’ handgrip strength was 28.8 ± 7.5 kg compared with 40.3 ± 8.0 kg in cisgender men. In contrast, in one cross‐sectional study comparing TM persons with cisgender women, handgrip strength was higher in TM persons (37.0 ± 6.0 kg) than in cisgender women (29.0 ± 5.0 kg) after 9.9 years of GAHT.^37^

#### 3.5.2 Feminizing GAHT

##### Prospective studies

Muscle strength decreased significantly in three out of six prospective studies in TF persons after initiating feminizing GAHT,^4^, ^35^, ^41^ and lower body isometric muscle strength remained unchanged after 12 months and 5 years of GAHT^27^, ^38^ in two out of six prospective studies (Table 1). Handgrip strength was unchanged during 12 months of GAHT in one out of six prospective studies.^31^ In Scharff (2019),^4^ handgrip strength decreased 4.3% after 12 months, with 67% of the decrease occurring in the last 3 months. In Yun (2021),^41^ handgrip strength decreased in 6.2% in the left and 7.7% in the right hand, but the strength decrease was only significant in the dominant right hand.

##### Cross‐sectional studies

In three cross‐sectional studies, comparing TF persons on GAHT with cisgender women, knee and elbow extension/flexion strength was similar between the two groups,^43^ while handgrip strength were higher in TF persons compared with cisgender women (Table 1).^32^, ^39^ In Hamilton (2024),^32^ the trained TF persons’ handgrip strength in their dominant hand was 40.7 ± 6.8 kg compared with 34.4 ± 3.8 kg in trained cisgender women, but TF persons had similar handgrip strength in their non‐dominant hand compared with cisgender women. In Alvares (2022),^39^ TF persons’ handgrip strength was 35.3 ± 5.4 kg compared with 29.7 ± 3.6 kg in cisgender women. In three cross‐sectional studies, comparing TF persons with cisgender men, TF persons had lower knee and elbow extension/flexion strength^43^ and handgrip strength compared with cisgender men.^36^, ^39^ Cutoff values for sarcopenia were evaluated in TF persons compared with cisgender men/women in one study.^34^ TF persons’ handgrip strength was 26.3 kg in the right hand and 24.8 kg in the left hand. In TF persons, muscle strength approached the threshold for sarcopenia in cisgender men (<27.0 kg), whereas muscle strength was above the sarcopenia threshold value for cisgender women (<16.0 kg).^34^ One cross‐sectional study compared TF persons who had undergone orchiectomy (*n* = 15) to TF persons on cyproterone acetate (*n* = 15), and found no significant difference in muscle strength between the two groups.^43^

### 3.6 Physical activity

Physical activity was assessed in five out of eight prospective studies^27^, ^31^, ^33^, ^35^, ^38^ and was unchanged after GAHT in all studies. One of the five prospective studies found a negligible increase in sports index (11.7%), leisure time (7.6%), work index (5.2%), and total physical activity (6.2%) over 12 months in TM persons.^33^ In one prospective study,^35^ TF persons were compared with cisgender men, and TF persons were significantly less physically active than cisgender men. In three out of three cross‐sectional studies on masculinizing GAHT,^32^, ^37^, ^40^ physical activity was comparable between TM persons and cisgender men and women. In one out of four cross‐sectional studies on feminizing GAHT,^36^ TF persons were significantly less physically active in sports‐related activities than cisgender men, while there was no difference between groups in the last three out of four cross‐sectional studies.^32^, ^39^, ^43^

## 4 DISCUSSION

The present review assessed published literature on muscle strength changes during masculinizing and feminizing GAHT. Fifteen studies were included, comprising data from 1206 transgender persons (722 TM persons and 484 TF persons). Overall, muscle strength appeared to increase during masculinizing GAHT and to decrease during feminizing GAHT. Physical activity was unchanged during GAHT and was comparable between TM persons and cisgender men or women, while TF persons were less physically active compared with cisgender men. Included studies were rated with moderate to high risk of bias. Thus, precaution is needed when interpreting the results.

Overall, masculinizing GAHT increased muscle strength in four out of six prospective studies. TM individuals demonstrated a 12 and 26% increase in isometric knee extension and flexion strength, respectively,^27^ and a 16–18% increase in upper body muscles^4^, ^33^ following 12 months of masculinizing GAHT. In cross‐sectional studies (mean GAHT duration ranging from 2 to 10 years), muscle strength was higher in TM persons compared with cisgender women, but lower compared with cisgender men. The findings of the present systematic review on masculinizing GAHT and muscle strength changes are in accordance with a previous narrative review from 2023.^49^ However, the present systematic review included four additional studies on masculinizing GAHT: two studies with prospective and two studies with cross‐sectional study design.^32^, ^37^, ^38^, ^42^ Data on long‐term GAHT duration of 6^38^ and 10 years^42^ were presented in two of these studies, which enabled us to review the prolonged effect of masculinizing GAHT on muscle strength.

In prospective studies, muscle strength increased significantly in the first year after initiating masculinizing GAHT.^4^, ^27^, ^33^, ^38^ Extended GAHT duration resulted in a small additional increase (6%) in lower body muscle strength between 1 and 6 years follow‐up,^27^, ^38^ whereas handgrip strength stabilized after 1 year of GAHT.^42^ Accordingly, a study in cisgender hypogonadal men on 3 years testosterone replacement therapy found that muscle strength increased after treatment initiation and then became stable after the first year.^50^ Likewise, a meta‐analysis in cisgender hypogonadal men on testosterone replacement therapy for 6–36 months^8^ did not find a significant association between treatment duration and muscle strength outcomes, indicating that longer treatment duration did not add further increase in muscle strength. These data support that muscle strength adaption mainly occurs in the beginning of masculinizing GAHT.

Handgrip strength (seven out of nine studies) or lower body strength (two out of nine) were study outcomes in the included studies.^4^, ^27^, ^31^, ^32^, ^33^, ^37^, ^38^, ^40^, ^42^ Notably, androgen receptors are more densely expressed in upper body muscles compared with lower body muscles,^51^ suggesting that masculinizing GAHT could have a more pronounced effect on upper body musculature compared with lower body muscles. In support of this hypothesis, in a frog model, muscle fibers in the shoulder region were more sensitive to testosterone than muscle fibers in other regions.^52^ Hence, a superior adaption in handgrip strength compared with lower body muscles during masculinizing GAHT could be expected. However, results regarding regional differences of muscle strength adaption in the present systematic review appeared inconclusive as two out of four prospective studies showed increased handgrip strength during masculinizing GAHT, while two out of two prospective studies showed increased muscle strength in the legs during masculinizing GAHT. This could be explained by the small muscle mass and high everyday activity pattern of the hand and lower arm^53^, ^54^ limiting GAHT‐mediated strength adaption. Therefore, physiological changes in muscle strength during GAHT may not directly translate into changes in handgrip strength,^55^ and future studies on GAHT and muscle function should involve the larger muscle groups (e.g., chest and/or back muscles) to fully evaluate the isolated GAHT‐induced muscle strength adaptions.

In cross‐sectional studies, muscle strength in TM persons using GAHT was lower compared with cisgender men,^32^, ^40^ but higher than cisgender women.^37^ Importantly, height and skeletal size do not adapt to masculinizing GAHT when initiated after puberty,^2^ and TM persons will most often have a smaller stature than cisgender men.^15^, ^56^ Height and skeletal muscle mass are linearly related^57^ and therefore TM persons receiving GAHT are unlikely to achieve muscle strength comparable to that of cisgender men. It could be hypothesized that muscle strength adaptions during masculinizing GAHT diminishes with increasing age, as sarcopenia becomes more prevalent with ageing.^58^ One study investigated changes in handgrip strength after initiating masculinizing GAHT between different age‐groups (<25, 25–40, and >40 years old)^4^ and reported no significant differences between age‐groups. The study included 278 TM persons, but the number of participants in different age‐groups was not specified. However, the mean age in the >40 years group was 47 years, and the reliability of this finding should be investigated further in studies involving larger muscle groups, preferably in the upper body, and studies including TM persons at higher age.

Feminizing GAHT decreased handgrip strength 6–8% after 6–12 months of GAHT in three out of six prospective studies,^4^, ^35^, ^41^ while muscle strength in the lower body muscles and handgrip strength were maintained in two out of six prospective studies^27^, ^38^ and one out of six prospective studies,^31^ respectively. Long‐term GAHT duration (from 1 to 5 years) did not change muscle strength in the lower body muscles.^38^ In most cross‐sectional studies (mean GAHT duration ranging from 3.1 to 14.4 years), muscle strength was significantly higher in TF persons compared with cisgender women, but lower compared with cisgender men.^36^, ^39^ It is important to notice, that only adult TF persons (mean age ranging from 27 to 41 years old) were included in the present review, which implied completion of male puberty. In accordance, TF persons who complete male puberty will have larger and longer bones than cisgender women,^56^ which would contribute to higher LBM^43^ and muscle strength than cisgender women.^15^, ^59^ Testosterone levels in TF persons were suppressed to the normal range of cisgender women, but the observed decrease in muscle strength after initiating feminizing GAHT was lower than the increase in muscle strength during masculinizing GAHT. These findings support that estradiol protects against loss of muscle strength.^16^, ^60^ Furthermore, cyproterone acetate was used as antiandrogen in most studies.^4^, ^31^, ^34^, ^35^, ^36^, ^39^, ^41^ Cyproterone acetate acts as a synthetic progesterone and could also be protective for muscle function.^61^ One cross‐sectional study^43^ found comparable muscle strength in upper and lower limbs between TF persons with orchiectomy (mean GAHT duration 8.7 years), TF persons without orchiectomy but receiving cyproterone acetate (mean GAHT duration 10.1 years), and cisgender women, but muscle strength in TF persons were lower than cisgender men. The study size was limited (*n* = 30 TF), and numerical muscle strength values were higher in TF persons compared with cisgender women,^43^ which suggested lack of study power. Progesterone is requested by some TF persons to improve mental health, but progesterone substitution is not part of routine transgender care in most countries due to lack of evidence.^2^, ^62^ Future studies are needed regarding the optimal use of antiandrogens to preserve muscle strength in TF persons.

Physical activity was unchanged in TM and TF persons during GAHT in the prospective studies, which indicated that muscle strength changes during GAHT was not primarily driven by parallel changes in physical activity. In cross‐sectional studies, physical activity was comparable between TM persons and cisgender men and women,^32^, ^37^, ^40^ while physical activity was comparable between TF persons and cisgender men and women in two out of four studies,^39^, ^43^ but TF persons were less physically active than cisgender men in two out of four studies.^35^, ^36^ Included studies regarding physical activity in the present systematic review should include muscle strength outcomes and limited sample sizes and low statistical power could explain findings of unchanged physical activity. In addition, the unchanged physical activity during feminizing GAHT could be caused by TF persons not wanting to appear masculine in their physical appearance.^63^ A recent systematic review^64^ on physical activity in transgender populations reported that transgender persons were less physically active than cisgender persons. However, the review was inconclusive on GAHT in transgender persons, which made it difficult to conclude on the possible role of GAHT on physical activity. A matched case–control study between transgender persons and cisgender men/women (matched for age and gender identity) found that both GAHT‐naïve transgender persons and transgender persons on GAHT engaged significantly less in physical activity compared with cisgender men/women, but within TF persons, physical activity levels were comparable to cisgender women.^23^ Furthermore, transgender persons on GAHT (*n* = 102) were more physically active compared with GAHT‐naïve transgender persons (*n* = 241), which could imply that GAHT may contribute to more engagement in physical activity. Improvement in mental health and higher body satisfaction during GAHT could be an explanation for higher physical activity.^1^ Engagement in physical activity has been associated with a higher quality of life among TM persons, but not among TF persons.^65^ This discrepancy could partially explain why physical activity were comparable between TM persons and cisgender persons in cross‐sectional studies, whereas TF persons appeared to be less physically active than cisgender men.^65^ It is possible that TM persons may be more motivated to exercise in order to achieve a more masculine body shape.^66^

TF persons undergoing feminizing GAHT are at high risk of losing muscle mass and muscle strength, and the GAHT‐mediated changes in body composition could result in insulin resistance,^67^ both factors contribute to a higher risk of developing cardiometabolic diseases. Furthermore, reduced muscle mass has been associated with metabolic syndrome.^68^ Physical activity prevents cardiometabolic diseases, why an active lifestyle is favorable to reduce the risk of cardiometabolic diseases.^69^ Masculinizing GAHT increases LDL‐cholesterol and triglycerides levels and lowers HLD‐cholesterol,^70^ however, the increase in muscle strength during masculinizing GAHT could protect against development of metabolic diseases.^68^ Future studies should investigate how to implement methods for transgender persons to engage in strength training or physical activity to stabilize muscle strength, especially during feminizing GAHT. Other lifestyle factors such as smoking cessation, weight management, and vitamin D supplementation must also be given attention.^71^

Both strengths and limitations should be considered in this study. Strengths of this study include the use of PRISMA guidelines, ensuring reproducibility, despite variations in study design (prospective vs. cross‐sectional), outcome measure assessment (lower limbs vs. elbow flexion/extension vs. handgrip strength), and follow‐up duration. Small study sample sizes with short follow‐up duration and lack of proper or sufficient control groups in the included studies are limitations that could affect the results. Small sample sizes did not allow for subgroup analysis according to different masculinizing/feminizing GAHT modalities, and pooling of different GAHT modalities and application forms into the same intervention group could result in type 1 error due to variation in measurement of muscle strength changes at follow‐up. Use of GnRH‐a before baseline in GAHT‐naïve transgender persons could cause lower baseline muscle mass and result in higher increase in muscle strength after GAHT initiation. Five studies were part of the ENIGI cohort,^4^, ^31^, ^33^, ^35^, ^37^ which could infer double presentation of results from some transgender participants. However, the results from these studies were consistent with other studies included in this review. The results cannot be generalized to North America, Oceania, and Africa, as none of the studies were conducted in these continents. An interobserver variability in the muscle strength test could be present, as there was no description of who performed the muscle testing. Furthermore, blinding of muscle tests regarding GAHT duration and GAHT modality was not addressed in the included studies, and knowledge of previous strength test results could have affected study outcomes. Physical activity was primarily self‐reported, introducing a risk of recall and social desirability biases, potentially leading to an over‐ or underestimation of physical activity, ultimately affecting the validity of the data.^72^ The IPAQ short form (IPAQ‐SF) was shown to overestimate physical activity and the evidence to use this questionnaire was weak.^73^ The Baecke questionnaire was applied in other studies,^31^, ^33^, ^35^, ^36^, ^37^ but this questionnaire had low sensitivity and specificity against accelerometer‐measured physical activity in participants with low education level.^74^ Studies measuring physical activity assessed objectively through wearable monitoring activity patterns could overcome the issue.^75^ Future studies with larger sample sizes, more detailed descriptions of outcome assessment, and control groups with GAHT‐naïve transgender persons are warranted to investigate muscle strength changes during GAHT. Differentiation between GAHT administration methods (e.g., transdermally vs. intramuscular injections), GAHT doses, and age at GAHT initiation in future studies are also warranted to better understand the impact on muscle strength and to optimize therapy strategies.

## 5 CONCLUSION

Masculinizing gender‐affirming hormone therapy appeared to increase muscle strength and feminizing gender‐affirming hormone therapy decreased muscle strength. Muscle strength was higher among transgender men/women compared with cisgender women, but lower compared with cisgender men. Physical activity was unchanged during gender‐affirming hormone therapy. Future studies should investigate how to engage transgender persons in physical activity to reduce the risk of cardiometabolic diseases during long‐term gender‐affirming hormone therapy.

## AUTHOR CONTRIBUTIONS

Mathilde Kamp NØrlund, Dorte Glintborg, and Louise Lehmann Christensen designed the review. Mathilde Kamp NØrlund conducted the search‐string with support from Tine Taulbjerg Kristensen, Mathilde Kamp NØrlund, and Jakob Lindberg Nielsen screened the articles and extracted data. Dorte Glintborg and Louise Lehmann Christensen supervised the process. All authors provided critical feedback, read, and approved the manuscript.

## CONFLICT OF INTEREST STATEMENT

The authors declare no conflict of interest.

## APPENDIX A

### Medline (Ovid)

(exp “Transgender Persons”/ OR transgend*.mp. OR exp “Transsexualism”/ OR transsex*.mp. OR exp “Gender Dysphoria”/ OR gender dysphor*.mp. OR “Female to male”.mp. OR FtM.mp. OR “Male to female”.mp. OR MtF.mp. OR Transvesti*.mp. OR “Sex chang*”.mp. OR Transfem*.mp. OR Transmasc*.mp. OR exp Psychosexual Development/ OR “Psychosexual development”.mp. OR “trans pe*”.mp. OR “sexual dysphoria”.mp. OR “sex transformation”.mp. OR “gender nonconform*”.mp. OR “Gender incongruence”.mp. OR nonbin*.mp. OR “non‐bin*”.mp.) AND (exp “Testosterone”/ OR “Testosterone”.mp. OR exp “Hormone Replacement Therapy”/ OR “hormone therap*”.mp. OR hormon*.mp. OR exp “Hormones”/ OR exp “Androgens”/ OR “Androgen*”.mp. OR exp “Progesterone”/ OR “Progesterone”.mp. OR exp “Estrogens”/ OR “Estrogen*”.mp. OR “Gender affirming hormone therap*”.mp. OR “Gender‐affirming hormone therap*”.mp. OR GAHT.mp.) AND (exp “Muscle Strength”/ OR “Muscle Strength”.mp. OR “muscular strength”.mp. OR exp “Exercise”/ OR “physical activity*”.mp. OR “physical activ*”.mp. OR “body strength”.mp. OR “limb strength”.mp. OR “handgrip strength”.mp. OR “hand‐grip strength”.mp.)

### Embase (Ovid)

(exp “Transgender Persons”/ OR transgend*.mp. OR exp “Transsexualism”/ OR transsex*.mp. OR exp “Gender Dysphoria”/ OR gender dysphor*.mp. OR “Female to male”.mp. OR FtM.mp. OR “Male to female”.mp. OR MtF.mp. OR Transvesti*.mp. OR “Sex chang*”.mp. OR Transfem*.mp. OR Transmasc*.mp. OR exp Psychosexual Development/ OR “Psychosexual development”.mp. OR “trans pe*”.mp. OR “sexual dysphoria”.mp. OR “sex transformation”.mp. OR “gender nonconform*”.mp. OR “Gender incongruence”.mp. OR nonbin*.mp. OR “non‐bin*”.mp.) AND (exp “Testosterone”/ OR “Testosterone”.mp. OR exp “Hormone Replacement Therapy”/ OR “hormone therap*”.mp. OR hormon*.mp. OR exp “Hormones”/ OR exp “Androgens”/ OR “Androgen*”.mp. OR exp “Progesterone”/ OR “Progesterone”.mp. OR exp “Estrogens”/ OR “Estrogen*”.mp. OR “Gender affirming hormone therap*”.mp. OR “Gender‐affirming hormone therap*”.mp. OR GAHT.mp.) AND (exp “Muscle Strength”/ OR “Muscle Strength”.mp. OR “muscular strength”.mp. OR exp “Exercise”/ OR “physical activity*”.mp. OR “physical activ*”.mp. OR “body strength”.mp. OR “limb strength”.mp. OR “handgrip strength”.mp. OR “hand‐grip strength”.mp.)

### PubMed

((“Transgender Persons”[Mesh] OR transgend* OR “Transmasc*” OR “Transfem*” OR “Transsexualism”[Mesh] OR transsexual* OR “Gender Identity”[Mesh] OR “gender identity” OR “Gender Dysphoria”[Mesh] OR “gender dysphoria” OR “Female to male” OR FtM OR Transvestism OR “Sex chang*” OR “Psychosexual Development”[Mesh] OR “Psychosexual Development” OR “Gender‐Variant” OR “sexual dysphoria” OR “sex inversion” OR “sex reversal” OR “sex alteration” OR “sex conversion” OR “sex transformation” OR “Sexual and Gender Minorities”[Mesh] OR “gender nonconform*” OR “nonbin*” OR “non‐bin*”) AND (“Testosterone”[Mesh] OR Testosterone OR “Hormone Replacement Therapy”[Mesh] OR “Hormones, Hormone Substitutes, and Hormone Antagonists”[Mesh] OR Androgen* OR “Androgens”[Mesh] OR “Anabolic Agents”[Mesh] OR anaboli* OR “Hormones”[Mesh] OR Hormones OR “Progesterone”[Mesh] OR Progesterone OR “Gonadal Steroid Hormones”[Mesh] OR “Estrogens”[Mesh] OR Estrogens OR “Gender‐affirming hormone therap*” OR “hormone treat*” OR GAHT OR “cross‐sex hormone*” OR “sex‐hormone treatment” OR “sex‐hormone therap*” OR “Gender affirming hormone therap*”) OR “feminizing” OR “masculinizing” AND (“Muscle Strength”[Mesh] OR “muscle strength” OR “Muscle Strength Dynamometer”[Mesh] OR “muscle strength dynamometer” OR “Hand Strength”[Mesh] OR “hand strength” OR “Handgrip Strength” OR “Hand‐grip strength” OR “Grip Strength” OR “physical activi*” OR “Exercise”[Mesh] OR exercise))

### Web of Science

TS = (“transgend*” OR “transsex*” OR “gender dysphor*” OR “Female to male” OR “FtM” OR “Male to female” OR “MtF” OR “Transvesti*” OR “Sex chang*” OR “Transfem*” OR “Transmasc*” OR “Psychosexual development” OR “trans pe*” OR “sexual dysphoria” OR “sex transformation” OR “gender nonconform*” OR “Gender incongruence” OR “nonbin*” OR “non‐bin*”) AND TS = (“Testosterone” OR “hormone therap*” OR hormon* OR “Androgen*” OR “Progesterone” OR “Estrogen*” OR “Gender affirming hormone therap*” OR “Gender‐affirming hormone therap*” OR “GAHT”) AND TS = (“Muscle Strength” OR “muscular strength” OR “Exercise” OR “physical activity*” OR “physical activ*” OR “body strength” OR “limb strength” OR “handgrip strength” OR “hand‐grip strength”)

## APPENDIX B

Newcastle‐Ottawa scale for cohort study

Selection (maximum 4 stars)

1. Representativeness of the exposed cohort
  - Truly representative of the average transgender person in the community (has to receive GAHT at a gender identity clinic)*
  - Somewhat representative of the average transgender person in the community*
  - Selected group of users, for example, nurses, volunteers
  - No description of the derivation of the cohort
2. Selection of the non‐exposed cohort
  - Drawn from the same community as the exposed cohort (e.g., transgender persons connected to a gender identity clinic, on waiting list to start GAHT)*
  - Drawn from a different source
  - No description of the derivation of the non‐exposed cohort
3. Ascertainment of exposure
  - Secure record (e.g., surgical records OR measurement of sex‐hormone levels to determine if they had been on GAHT)*
  - Structured interview*
  - Written self‐report
  - No description
4. Demonstration that outcome of interest was not present at start of study
  - Yes (measurements of outcome at baseline/prior to therapy)*
  - No

Comparability (maximum 2 stars)

1. Comparability of cohorts on the basis of the design or analysis
  - Study controls for age*
  - Study controls for BMI, physical activity, socioeconomic status*

Outcome (maximum 3 stars)

1. Assessment of outcome
  - Independent blind assessment*
  - Record linkage*
  - Self report
  - No description
  - Other
2. Was follow‐up long enough for outcomes to occur
  - Yes >3 months*
  - No <3 months
3. Adequacy of follow up of cohorts
  - Complete follow up—all participants accounted for*
  - Participants lost to follow up unlikely to introduce bias—small number lost— >80 % follow up, or description provided of those lost)*
  - Follow up rate <80% and no description of those lost
  - No statement

Newcastle‐Ottawa scale adapted for cross‐sectional studies

Selection (maximum 5 stars)

1. Representativeness of the exposed cohort
  - Truly representative of the average transgender person in the community (has to receive GAHT at a gender identity clinic)*
  - Somewhat representative of the average transgender person in the community*
  - Selected group of users, for example, nurses, volunteers
  - No description of the derivation of the cohort
2. Sample size
  - Justified and satisfactory (>30 transgender)*
  - Not justified (<30 transgender)
3. Non‐respondents
  - Comparability between respondents and non‐respondents characteristics is established, and the response rate is satisfactory*
  - The response rate is unsatisfactory, or the comparability between respondents and non‐respondents is unsatisfactory.
  - No description of the response rate or the characteristics of the responders and the non‐responders.
4. Ascertainment of exposure
  - Validated measurement tool** (measurement of sex‐hormone levels to determine if they had been on GAHT)
  - Non‐validated measurement tool, but the tool is available or described*
  - No description

Comparability (maximum 2 stars)

1. The participants in different outcome groups are comparable, based on the study design or analysis. Confounding factors are controlled.
  - Study controls for age*
  - Study controls for BMI, physical activity, socioeconomic status*

Outcome (maximum 3 stars)

1. Assessment of outcome
  - Independent blind assessment**
  - Record linkage*
  - Self report
  - No description
  - Other
2. Statistical test:
  - The statistical test used to analyze the data is clearly described and appropriate, and the measurement of the association is presented, including confidence intervals and the probability level (*p* value)*
  - The statistical test is not appropriate, not described or incomplete.
